# Supplementary material for: ATP7B-Deficient Hepatocytes Reveal the Importance of Protein Misfolding Induced at Low Copper Concentration
Source: Cells. 2022 Oct 27;11(21):3400. doi: 10.3390/cells11213400 (PMC9657033; doi:10.3390/cells11213400)
Supplement: Supplementary file 1 [file cells-11-03400-s001.zip › cells-1995196-supplementary.pdf]

Supplementary Information

ATP7B-deficient hepatocytes reveal the importance of protein misfolding induced at low copper concentration

Author list: Peggy Charbonnier[1], Benoît Chovelon[2,3], Corinne Ravelet[3], Tuan Dung Ngo[1], Mireille Chevallet[1] and Aurélien Deniaud[1,*]

Affiliations

[1] Univ. Grenoble Alpes, CNRS, CEA, IRIG - Laboratoire de Chimie et Biologie des Métaux, F-38000 Grenoble, France

[2] Service de Biochimie SB2TE, Institut de Biologie et Pathologie CHU Grenoble Alpes

[3] Département de Pharmacochimie Moléculaire, Université Grenoble Alpes, CNRS, UMR 5063, F-38041 Grenoble, France

* Correspondence should be addressed to Dr Aurélien Deniaud, aurelien.deniaud@cea.fr

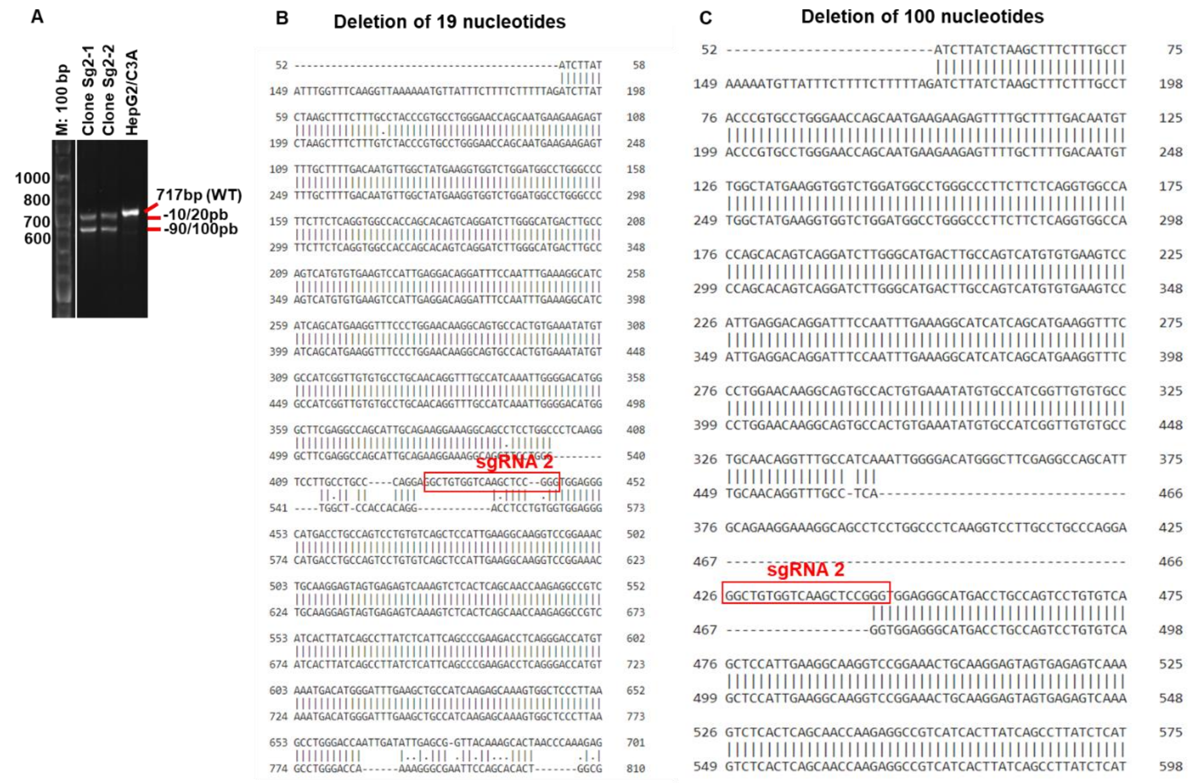

Figure S1. Analysis of CrispR/Cas9 clones obtained using the sgRNA-2. (**A**) An acrylamide gel electrophoresis of the PCR products obtained for sg2-1 and sg2-2 clones compared with those obtained with the HepG2/C3a parental cell line. While only one band was observed with HepG2/C3a cells corresponding to the WT version of *atp7b* for the two alleles, two different products were obtained for the two clones corresponding to a deletion of 10-20 nucleotides and a deletion of 90-100 nucleotides. Sequencing results for the Sg2-1 clone confirmed the presence of a 19 nucleotide deletion for the first allele (**B**) and a 100 nucleotide deletion for the second allele (**C**).

Primer designation	Primer sequence
Sg1-short-for	CCATGTGAGTGATAAGTGGCG
Sg1-short-rev	CTCACCTGTGATGAGGGACTC
Sg1-long-for	TGCAGCAGCCAAAGAGATCA
Sg1-long-rev	CCACCATATAGCCCAAGGCA
Sg2-short-for	CATCAAATTGGGGACATGGGC
Sg2-short-rev	CTCCTTGCAGTTTCCGGACC
Sg2-long-for	ACACCAGTGGCATTGTTTTCCA
Sg2-long-rev	TTGGTCCCAGGCTTAAGGGA
Sg3-for	CCTCCTCTCCCGGGACTTTA
Sg4-short-for	TGAAGGCAAGGTCCGGAAAC
Sg4-short-rev	CTCACCTGTGATGAGGGACTC
Sg4-long-for	CCAGAGAAGCTGGGATGTTGT
Sg4-long-rev and Sg3-rev	TCGGCTCCATCAGGAAGAGA

Table S1. Sequences of the primers used to amplify the regions surrounding the different sgRNA sequences.

Gene	Forward primer sequence	Reverse primer sequence
HPRT	ATGGACAGGACTGGACGTCTTGCT	TTGAGCACACAGAGGGCTACAATG
GAPDH	ATGGGGAAGGTGAAGGTCG	GGGGTCATTGATGGCAACAATA
Met (MT1X)	GCTTCTCCTTGCCTCGAA	TGACGTCCCTTTGCAGATG
GCLM	CCTCCTGCTGTGTGATGCCAC	CGTGCGCTTGAATGTCAGGAATGC
SOD1	TGGCCGATGTGTCTATTGAA	ACCTTT GCCAAGTCATCTG
Catalase	TTGCCACAGGAAAGTACCCC	TGAGGCCAAACCTTGGTGAG
HMOX1	ATGACACCAAGGACCAGAGC	GTGTAAGGACCCATCGGAGA
IL8	TGGCAGCCTTCCTGATTTCT	ATTTCTGTGTTGGCGCAGTG
HSPA6	AGGAGGTGGAGAGGATGGTT	TGTCCTCTTCGGGAATCTTG
DNAJ-A1	CTGCAACGGAAGGAAGATAGT	GTCTCCTTCACCATGGAATGT
DNAJ-A2	CAGGGTGTGTTCGTGTAGTT	GTCTGGGTTGATCCAGTTGT
DNAJ-A3	AGGACAAGCCAAGCAGAAA	TCTGCACCCTGAACGTAATG
ATG7	ACAGATGGAGTAGCAGTTTCC	ACATTCACTGAGGTTCACCAT
ATG13	TCCAGACAGTTCGTGTTGGG	CTCAAATTGCCTGGTAGACATGA
BNIP3L	CGCCCCTGCACAACAAC	TCATTGCCATTGCTGCTG
FUNDC1	TCAGATTGCTAGTCATAGTGGC	TGCTTTGTTCGCTCGTTTCT
Get3	TGAGCATGGGCAAGAAGATG	GTCAAATACCACCACCGAGAAG
PINK1	TGGTCGACTACCCTGATGTG	CGCAGGGTACAGGGATAGTT
Drp1	TCACCCGGAGACCTCTCATT	TCTGCTTCCACCCCATTTTCT
BSEP	ACATGCTTGCGAGGACCTTTA	GGAGGTTCGTGCACCAGGTA

Table S2. Sequences of the primers used for quantitative real time polymerase chain reaction.
